# MRI-based nomogram analysis: recognition of anterior peritoneal reflection and its relationship to rectal cancers

**DOI:** 10.1186/s12880-021-00583-7

**Published:** 2021-03-17

**Authors:** Shaoting Zhang, Fangying Chen, Xiaolu Ma, Minjie Wang, Guanyu Yu, Fu Shen, Xianhua Gao, Jianping Lu

**Affiliations:** 1 Department of Radiology, Changhai Hospital, 168 Changhai Road, Shanghai, 200433 China; 2 Department of Colorectal Surgery, Changhai Hospital, 168 Changhai Road, Shanghai, 200433 China

**Keywords:** Magnetic resonance imaging, Anterior peritoneal reflection, Rectal cancer, Nomogram

## Abstract

**Background:**

This study is aimed to explore the factors influencing the visualization of the anterior peritoneal reflection (APR) and evaluated the feasibility of measuring the distance from the anal verge to APR (AV-APR), the tumor height on MRI and the accuracy of determining the tumor location with regard to APR.

**Methods:**

We retrospectively analyzed 110 patients with rectal cancer. A univariate and multivariate logistic regression was performed to identify the independent factors (age, sex, T stage, the degree of bladder filling, pelvic effusion, intraoperative tumor location, BMI, uterine orientation, the distance from seminal vesicle/uterus to rectum) associated with the visualization of the APR on MRI. The nomogram diagram and receiver operating characteristic curve (ROC curve) were established. Intraclass correlation coefficient (ICC) was used to evaluate the consistency of the distance of AV-APR. The Pearson correlation coefficient was used to characterize the agreement between measurements of the tumor height by colonoscopy and MRI. The Kappa statistics was used to evaluate the value of MRI in the diagnosis of the tumor location with regard to the APR.

**Results:**

Multivariate logistic regression showed that BMI (*P* = 0.031, odds ratio, OR = 1.197), pelvic effusion (*P* = 0.020, OR = 7.107) and the distance from seminal vesicle/uterus to the rectum (*P* = 0.001, OR = 3.622) were correlated with the visualization of APR. The cut-off point of BMI and the distance from seminal vesicle/uterus to the rectum is 25.845 kg/m^2^ and 1.15 cm. The area under curve (AUC) (95% Confidence Interval, 95% CI) of the combined model is 0.840 (0.750–0.930). The favorable calibration of the nomogram showed a non-significant Hosmer–Lemeshow test statistic (*P* = 0.195). The ICC value (95% CI) of the distance of AV-APR measured by two radiologists was 0.981 (0.969–0.989). The height measured by MRI and colonoscopy were correlated with each other (r = 0.699, *P* < 0.001). The Kappa value was 0.854.

**Conclusions:**

BMI, pelvic effusion, and the distance from seminal vesicle/uterus to rectum could affect the visualization of APR on MRI. Also, it’s feasible to measure the distance of AV-APR, the tumor height, and to evaluate the tumor location with regard to APR using MRI.

**Supplementary Information:**

The online version contains supplementary material available at 10.1186/s12880-021-00583-7.

## Background

Colorectal cancer (CRC) is one of the most common gastrointestinal cancers, with rectal cancer (RC) accounting for 30–35%. According to the reported data, CRC ranks third in incidence and second in mortality worldwide [1]. Over the past decade, the incidence of CRC has rapidly declined in the wake of widespread colonoscopy uptake in developed countries. However, the decline in the overall CRC incidence rate masked an increase of 2% per year among adults younger than 55 years that have been recorded over recent years [2, 3]. The peritoneum covers the anterior wall of the upper rectum, whereas the middle and lower thirds lie below the peritoneal reflection and are completely encircled by mesorectum [4]. Above and below the anterior peritoneal reflection (APR), the lymphatic spread of cancer is inconsistent [5, 6]. Under the APR, they are mainly drained through the lateral lymph, while above the APR, they are mainly drained to the inferior mesentery. And according to the location and stage of the tumor, the treatment and prognosis of rectal cancer may significantly differ [7, 8]. Some surgeons propose that the APR could be a suitable landmark for identifying patients with rectal cancer for radiation. And the overall reported 5-year local recurrence rate for intraperitoneal and extraperitoneal rectal cancer is 4.2% and 13.3%, respectively [9]. Therefore, accurate recognition of the APR and the tumor location with regard to APR before the operation is useful in choosing the appropriate treatment strategies so as to avoid under or over-treatment [10–14]. In previous studies, rigid endoscopy or intraoperative proctoscopy were used to measure the distance of AV-APR [15, 16]. Still, the results were highly variable, and the tumor location towards the APR could not be accurately evaluated.

**Fig. 1:**
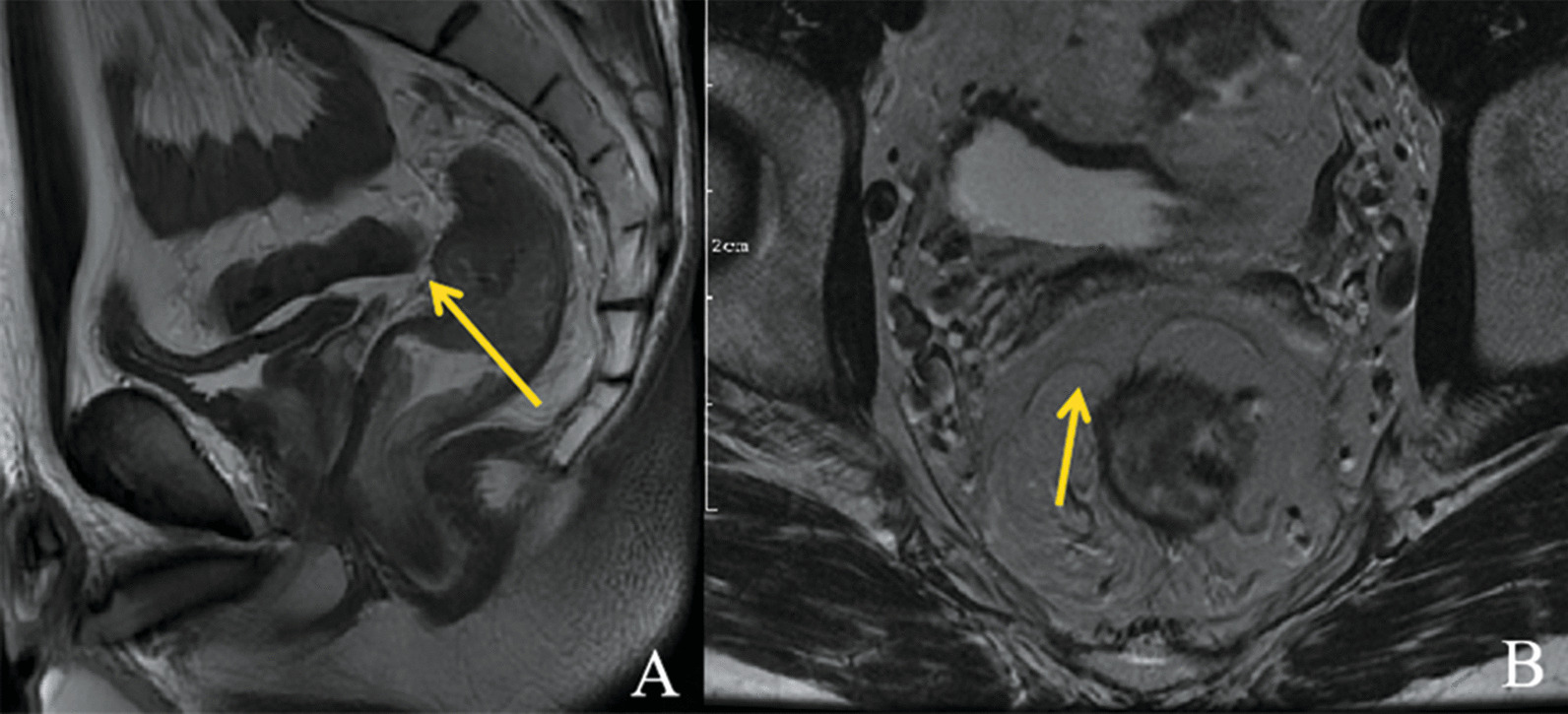
Identification of the APR on MR imaging. **a** Sagittal plane: the APR was a thin hypointense linear structure noted along with the superior bladder, which extended inferiorly and posteriorly to the tip of the seminal vesicles, after which the posterior extension attached to the anterior rectal wall. **b** Axial plane: the APR attaches to the anterior rectal wall in a V-shaped hypointense configuration. APR: anterior peritoneal reflection

Magnetic resonance imaging (MRI) has a good soft tissue resolution and can also identify APR [17–19]. On the axial T2-weighted (T2W) images, APR shows a V-shaped hypointense configuration attached to the anterior rectal wall. However, the axial images cannot precisely reveal the distance of AV-APR. On the sagittal T2W images, APR was identified as a thin hypointense linear structure noted along with the superior bladder (men) or uterus (women), which extended inferiorly and posteriorly to the tip of the seminal vesicles in men and to the cul-de-sac in women, after which the posterior extension attached to the anterior rectal wall (Fig. 1a, b) [20]. Unfortunately, not all APRs can be visualized on MRI in clinical practice [4].

Therefore, we explored the factors that affect the visualization of the APR on sagittal MRI. Moreover, we assessed the feasibility of measuring the distance of AV-APR and the height of the tumor using MRI, as well as the accuracy of MRI for recognizing the tumor location with regard to the APR.

## Methods

### Patients

All methods of the present research were carried out in accordance with the Declaration of Helsinki and were approved by the local Institutional Review Board (Committee on Ethics of Biomedicine, Changhai Hospital, Shanghai, China). Informed consent was waived for this retrospective study. A total of 320 consecutive patients diagnosed with rectal cancer and then treated in our hospital between January 2019 and June 2019 were enrolled in this retrospective study. Selection criteria included the following: ① a biopsy-confirmed primary rectal carcinoma, ② treatment by surgical resection, ③ a preoperative rectal MRI with good image quality, ④ operation within two weeks after MRI. Exclusion criteria included the following: ① patients with MRI images with motion artifacts or poor image quality (n = 30), ② patients who received radiotherapy, neoadjuvant treatment or/and palliative treatment (n = 100), ③ an interval between MRI and surgery higher than 2 weeks (n = 20), ④ patients with the previous history of other pelvic surgery (n = 60).

The collected clinical and imaging data included the following: patient age, sex, Body Mass Index (BMI), T stage, the tumor location with regard to APR (MRI and intraoperative findings), the degree of bladder filling, the orientation of the uterus, pelvic effusion, and the distance from seminal vesicle/uterus to the rectum, the height of tumor (MRI and colonoscopy), and the distance of AV-APR.

### MRI-sequence acquisition

MRI was performed on a 3.0 Tesla (T) MRI scanner (MAGNETOM Skyra, Siemens Healthcare, Erlangen, Germany) using a phased-array body coil while patients were placed in a supine position. Before scanning, intestinal cleaning was performed by enema administration with 20 ml of glycerin. MRI scan sequences included: ① high-resolution oblique axial T2WI without fat saturation, ② sagittal T2WI (turbo-spin-echo, TSE) without fat saturation, ③ axial T1-weighted image (T1WI), ④ axial diffusion weighted images (b = 0, 1000 s/mm^2^), and ⑤ gadolinium contrast-enhanced T1WI with fat saturation (axial, sagittal and coronal planes). The scanning parameters of sagittal T2WI were as follows: repetition time/echo time [TR/TE]: 5000/106 ms, field of view [FOV]: 23 cm, section thickness: 5 mm, number of slices: 23 slices, voxel: 0.7 * 0.7 * 5.0 mm, bandwidth: 200 Hz/pixel, averages: 2, flip angle: 180, total time: 157 s.

### Radiologist and colorectal surgeons revaluation strategy and anatomic measurements

#### Radiologists

**Fig. 2:**
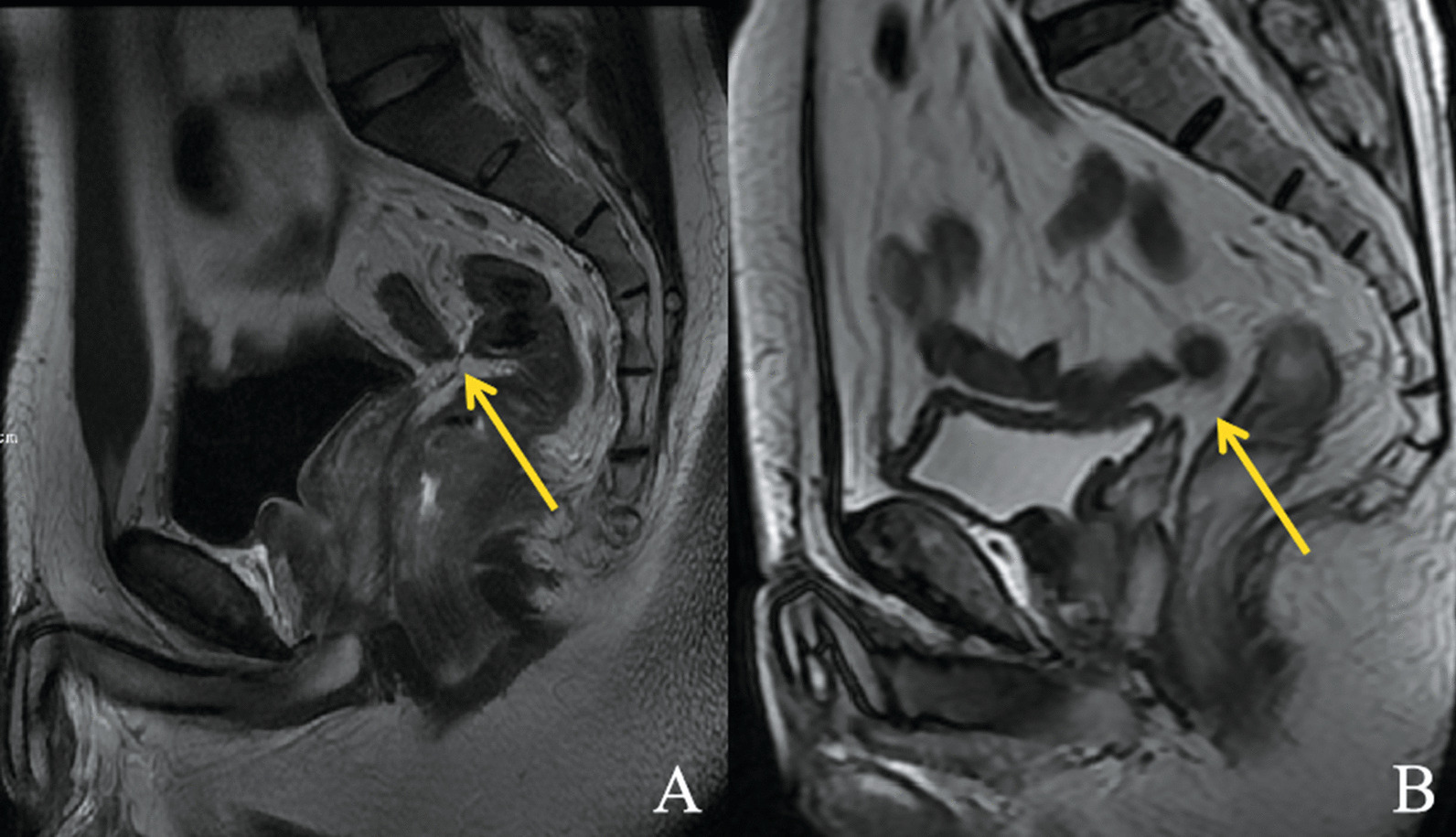
The visualization of the APR on sagittal T2W images. **a** APR was definitely visible. **b** APR was probably visible

**Fig. 3:**
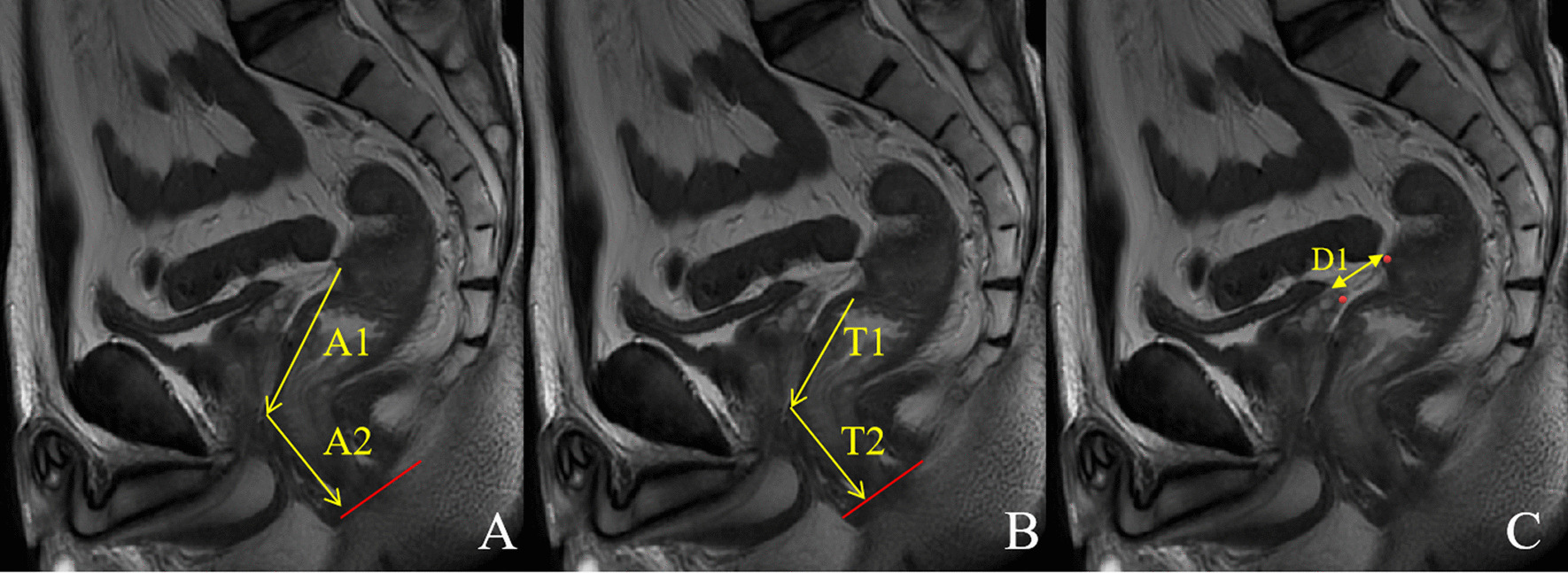
Measurement of various distances on sagittal T2W images. **a** The height of the APR from the anal verge as a line from the APR to the anal verge along the direction of the rectum: A1 + A2. **b** The tumor height from the anal verge was measured as the distance from the inferior tumor margin to the anal verge: T1 + T2. **c** The distance from the seminal vesicle to the rectum was measured as a line from the junction of APR and seminal vesicle to the junction of APR and rectum along the direction of APR: D1

MR images were independently reviewed by two gastrointestinal radiologists (SZ and FC) with more than 5 years' working experience in rectal MRI. The two readers were blinded to any pathological results of the patients and achieved a unified standard to evaluate the MRI features through discussion before the independent analysis. If there were discrepancies between the two readers on qualitative parameters, a final decision was reached by a third reader (FS with 11 years of experience in imaging diagnosis). The readers cross-referenced the oblique axial T2W and sagittal T2W imaging to recognize APR. We divided APR into two categories: definitely visible (the thin hypointense linear structure attached to the anterior rectal wall is definitely visible) and probably visible (the thin hypointense linear structure is probably visible or definitely not visible) (Fig. 2a, b) [19]. For all the patients rated with a definitely visible APR, the distances of AV-APR were measured in the sagittal T2W images as a line from the APR to the anal verge along the direction of the rectum (Fig. 3a) [4]. For all the patients, the height of tumor was measured as the distance from the inferior tumor margin to the anal verge (Fig. 3b). The distances from seminal vesicle/uterus to rectum were measured as a line from the junction of APR and seminal vesicle/uterus to the junction of APR and rectum along the direction of APR (Fig. 3c). The tumor location with regard to APR (MRI) was assigned to the following categories: ① above the APR (the distal end of the tumor reaches above the height of the APR), ② straddle the APR (the distal end of the tumor reaches below the height of the APR and the proximal end of the tumor reaches above the height of the APR), ③ below the APR (the proximal end of the tumor reaches below the height of the APR). According to the angle between the axis of the uterus and the axis of the axial plane on the sagittal T2WI, uterine orientation was categorized as follows: anteversion, perpendicular, or retroversion. A distended bladder was defined as the bladder wall showing no folds on both sagittal and axial T2WI [4], which means that the bladder was filling.

#### Colorectal surgeons

The two surgeons were blinded to any MRI results of the patients. For all the patients, the height of tumor was measured by colonoscopy. And the tumor location with regard to APR (intraoperative findings) was also assigned to the following categories: ① above the APR, ② straddle the APR, ③ below the APR. If there were discrepancies between the two reviewers, a final decision was reached by consensus.

### Statistical analysis

For continuous variables, the normality test was carried out by One-Sample Kolmogorov–Smirnov test. Data conforming to normal distribution were reported as mean ± standard deviation, and data not conforming to normal distribution were reported as median and quartile. The Chi-square test or Fisher’s exact test was carried out for assessing categorical variables, as appropriate. The Independent-Samples T test was carried out for assessing continuous variables. A univariate logistic regression analysis was performed to identify the independent factors associated with the visualization of the APR on MRI. Then, multivariate logistic regression combined the selected influencing factors was performed to establish a combined model. Moreover, the nomogram analysis and the receiver operating characteristic curve (ROC curve) were performed. Intraclass correlation coefficients (ICC) were used to evaluate the differences in the distance of AV-APR between the two radiologists. The Pearson correlation coefficient was used to characterize the agreement between pairs of measurements of the tumor height by colonoscopy and MRI. The consistency check of a diagnostic test (Kappa statistics) was used to evaluate the value of MRI in the diagnosis of the tumor location with regard to the APR. Statistical analyses were performed using SPSS version 19.0 for windows (SPSS Inc., Chicago, Illinois, USA) and R software (version 3.4.3). All *P* values < 0.05 were considered statistically significant.

## Results

### Clinical characteristics

A total of 110 patients were finally included in this study. The APR was “definitely visible” in 75 of 110 cases (68.2%) and “probably visible” in 35 of 110 cases (31.8%) (definitely not visible was 0). The tumor height measured by MRI, age, BMI, the distance of AV-APR conformed to normal distribution (*P* > 0.05), and the tumor height measured by colonoscopy, the distance from seminal vesicle/uterus to rectum didn’t conform to normal distribution (*P* < 0.05). The mean age was 60.22 ± 10.03 (range, 35–85) years. Other characteristics of patients are listed in Table 1. The two observers' agreement for objective parameters are listed in Additional file 1: Supplemental Table 1.

**Table 1:** Characteristics of patients with rectal cancer

Variables	No. of case/mean ± SD/median (interquartile range)			*P* value
	Total (N = 110)	APR with probably visible (N = 35)	APR with definitely visible (N = 75)	
Age (year)	60.22 ± 10.03	60.54 ± 8.60	60.07 ± 10.68	0.818
BMI (kg/m^2^)	24.33 ± 3.14	23.30 ± 3.12	24.81 ± 3.04	0.018
Distance of AV-APR (cm)	9.60 ± 1.22	NA	9.60 ± 1.22	NA
Distance from seminal vesicle/uterus to rectum (cm)	1.28 (0.85,1.81)	0.70 (0.52, 1.07)	1.55 (1.13, 2.00)	0.018
Height of tumor measured by MRI (cm)	6.89 ± 2.53	6.75 ± 2.26	6.94 ± 2.65	0.706
Height of tumor measured by colonoscopy (cm)	7.00 (5.00,10.00)	6.00 (5.00, 8.00)	7.00 (5.00,10.00)	0.288
Sex				
Male	65 (59.1%)	22 (20.0%)	43 (39.1%)	0.583
Female	45 (40.9%)	13 (11.8%)	32 (29.1%)	
Degree of bladder filling				
Filling	44 (40.0%)	10 (9.1%)	34 (30.9%)	0.095
Not-filling	66 (60.0%)	25 (22.7%)	41 (37.3%)	NA
Pelvic effusion				
Yes	19 (17.3%)	2 (1.8%)	17 (15.5%)	0.028
No	91 (82.7%)	33 (30.0%)	58 (52.7%)	NA
Orientation of uterus (N = 45)				
Anteversion	33 (73.3%)	9 (20.0%)	24 (53.3%)	0.692
Retroverted	12 (26.7%)	4 (8.9%)	8 (17.8%)	NA
T stage				
T1	7 (6.3%)	3 (2.7%)	4 (3.6%)	0.830
T2	23 (20.9%)	7 (6.4%)	16 (14.5%)	NA
T3	79 (71.8%)	25 (22.7%)	54 (49.1%)	NA
T4	1 (0.09%)	0 (0.0%)	1 (0.09%)	NA

APR, anterior peritoneal reflection; AV, anal verge; SD, standard deviation; BMI, body mass index

### Distance of AV- APR measured upon MRI and comparisons of tumor height measured by colonoscopy and MRI

There were 75 patients with definitely visible APRs, including 32 females and 43 males. The mean distance of AV-APR was 9.60 ± 1.22 cm in total, 9.57 ± 0.68 cm in females, 9.62 ± 1.47 cm in males (*P* = 0.857). The ICC value (95% Confidence Interval, 95% CI) of the distance measured by two radiologists was 0.981 (0.969–0.989). The mean height of the tumor measured by MRI was 6.89 ± 2.53 cm. The median and interquartile range of the height measured by MRI and colonoscopy were 7.05 (5.21–8.75) and 7.00 (5.00–10.00) cm, respectively. The two results were correlated with each other (r = 0.699, *P* < 0.001).

### Comparisons of tumor location with regard to the APR by MRI and by intraoperative findings

**Fig. 4:**
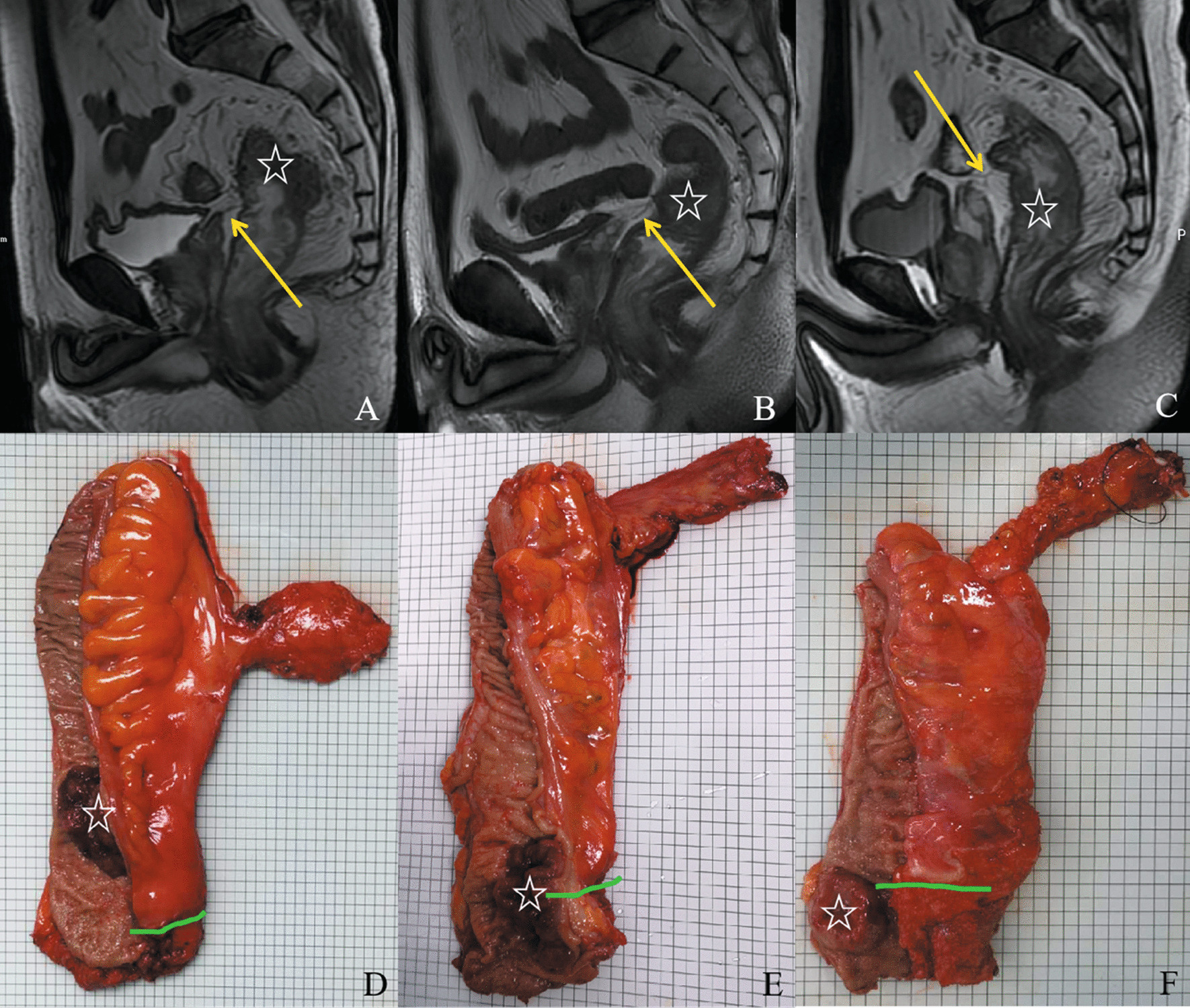
Tumor location with regard to the anterior peritoneal reflection (APR) was determined by MRI (**a**–**c**) and intraoperative palpation and visualization (**d**–**f**). “☆” in MRI imaging: tumor; yellow arrow in MRI imaging: APR; green line in resected specimens: APR. **a**, **d**: above the APR; **b**, **e**: straddle the APR; **c**, **f**: below the APR

The accuracy of the tumor locations with regard to the APR (determined via MRI) was 90.0% compared with intraoperative findings (Fig. 4, Table 2). The Kappa value of tumor location with respect to the APR determined by MRI and intraoperative findings was 0.854 (*P* < 0.001).

**Table 2:** Tumor location with regard to the APR by MRI and intraoperative findings

	By intraoperative findings			
	Above the APR	Straddle the APR	Below the APR	Total
By MRI				
Above the APR	18	2	3	23
Straddle the APR	0	34	6	40
Below the APR	0	0	47	47
Total	18	36	56	110
Accuracy rate	100% (18/18)	94.40% (34/36)	83.90% (47/56)	90.00% (99/110)

APR, anterior peritoneal reflection

### Factors influencing the visualization of the APR upon MRI

**Fig. 5:**
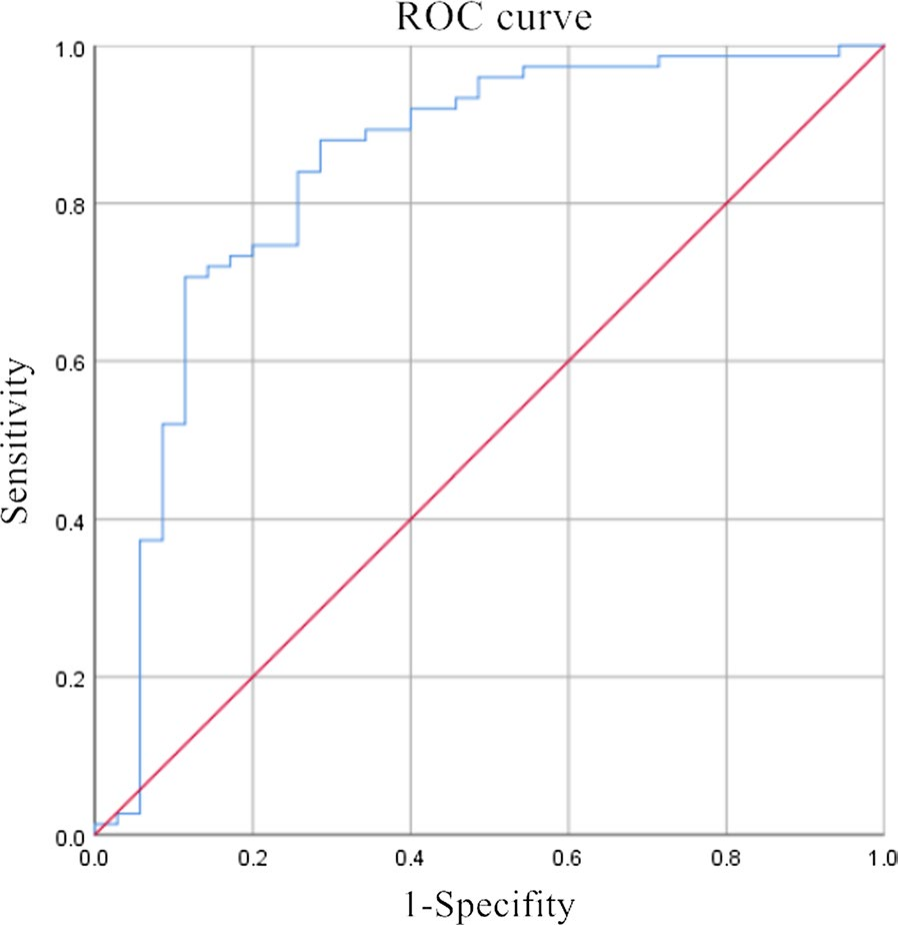
The ROC curve of the combined model. The area under curve (AUC) (95% CI) of the combined model is 0.840 (0.750–0.930), the sensitivity is 0.880 and the specificity is 0.714

**Fig. 6:**
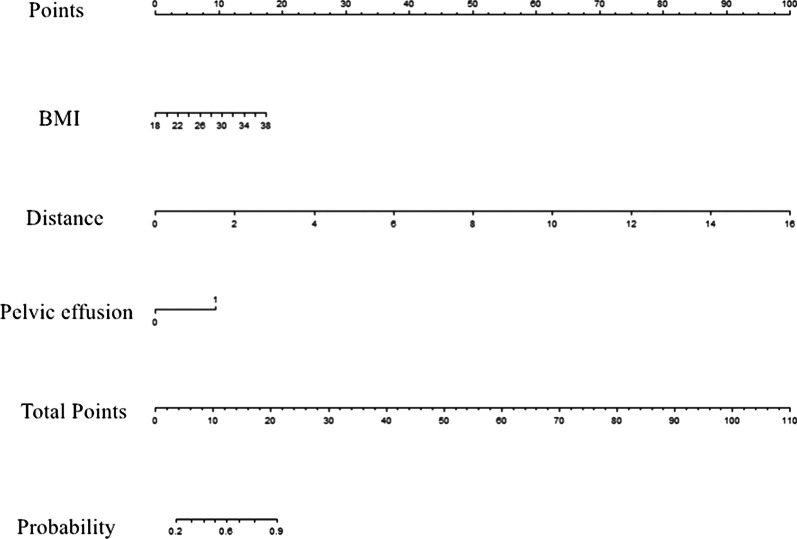
Nomogram of the combined model. In the nomogram, first, a vertical line was drawn according to the value of the three influencing factors label to determine the corresponding value of points. The total points were the sum of the three points above. Then, a vertical line was made according to the value of the total points to determine the probability of the visualization of APR

Factors influencing the visualization of the APR were BMI (*P* = 0.021), pelvic effusion (*P* = 0.043), and the distance from seminal vesicle/uterus to the rectum (*P* = 0.001), as determined by univariate analysis. Other factors, such as age, sex, T stage, the degree of bladder filling, the orientation of the uterus, and the location of the tumor did not influence the visualization of the APR (*P* > 0.05). A multivariate logistic regression analysis found that factors influencing the visualization of the APR were BMI (*P* = 0.031, odds ratio, OR = 1.197), pelvic effusion (*P* = 0.020, OR = 7.107) and the distance from seminal vesicle/uterus to the rectum (*P* = 0.001, OR = 3.622) (Table 3). The cut-off point of BMI and the distance from seminal vesicle/uterus to the rectum is 25.845 kg/m^2^ and 1.15 cm, respectively. We established the regression equation {Y = − 5.396 + 0.180 * BMI + 1.961 * [pelvic effusion (yes)] + 1.287 * distance from seminal vesicle/uterus to the rectum} and the ROC curve of the combined model. The area under curve (AUC) (95% CI) of the combined model is 0.840 (0.750–0.930), the sensitivity is 0.880 and the specificity is 0.714 (Fig. 5). A predictive nomogram was constructed based on the multivariate logistical regression combined with the selected factors to develop a prediction model for the visualization of the APR (Fig. 6). The favorable calibration of the nomogram showed a non-significant Hosmer–Lemeshow test statistic (*P* = 0.195).

**Table 3:** Logistic regression analyses of factors that affect the visualization of the APR on MRI

Variables	Univariate logistic regression		Multivariate logistic regression	
	OR (95% CI)	*P*	OR (95% CI)	*P*
Age	0.995 (0.956–1.036)	0.816	NA	NA
Sex	1.259 (0.552–2.872)	0.583	NA	NA
T stage	1.189 (0.621–2.276)	0.602	NA	NA
Degree of bladder filling	0.482 (0.204–1.143)	0.098	NA	NA
Pelvic effusion	4.836 (1.051–22.250)	0.043	7.107 (1.360–37.148)	0.020
Tumor location by intraoperative findings	0.827 (0.475–1.440)	0.502	NA	NA
BMI	1.192 (1.026–1.385)	0.021	1.197 (1.017–1.409)	0.031
Orientation of uterus	0.750 (0.181–3.115)	0.692	NA	NA
Distance from seminal vesicle/uterus to rectum	3.561 (1.692–7.493)	0.001	3.622 (1.642–7.990)	0.001

BMI, body mass index; CI, confidence interval; OR, odd ratio

## Discussion

In this study, we found that BMI, pelvic effusion, and the distance from seminal vesicle/uterus to rectum resulted in risk factors influencing the visualization of the APR at MRI. Sun et al. [4] found that the wider the space surrounding the APR, the easier it was to observe the APR, so we introduced the concept of the distance from seminal vesicle/uterus to the rectum, which provided the most direct reflection of the space around APR; the farther distance (> 1.15 cm) was associated with the larger space and clearer APR display. BMI is an important indicator of the degree of obesity in the human body, which indirectly reflects the size of the pelvic space. Patients with larger BMI (> 25.845 kg/m^2^) had more dispersed pelvic organs as well as larger space around the APR, which made it easier to observe the APR. Gollub found that APR was especially visible in cases where fluid was present in the pelvic cul-de-sac [19]. We also found that in patients with pelvic effusion, APR was shown more clearly. The effusion may appear at the lowest point of the pelvic cavity, which can contrast the APR. Sun et al. [4] have reported that the degree of bladder filling, the orientation of the uterus, and age are the factors that affect the visualization of the APR (283 of 319 was visible). However, we found no association between these variables and visualization of the APR. Nevertheless, we can increase the distance from seminal vesicle/uterus to rectum by minimizing the contents of the bladder, so as to improve the possibility of the visualization of the APR.

We found that the mean distance of AV-APR was 9.57±0.68 cm in females and 9.62±1.47 cm in male; the observed difference was not statistically significant, which was consistent with the results of Yun (the mean distance of AV-APR was 8.80±2.20 cm in females and 8.10±1.70 cm in male) [15]. However, Sun [4] reported the significant difference in sex, with the distance of 10.4±1.1 cm for females and 10.0±1.2 cm for males.

In this study, we also found that MRI can be used to evaluate the tumor location with regard to the APR. The accuracy was close to 90.0%. In previous studies, computed tomography (CT), and transrectal ultrasonography (TRUS) were also used to evaluate the distance and location relationship between APR and tumor, but they all had different disadvantages. CT could identify the rectal cancer location with regard to the APR, but the soft tissue resolution of CT is worse than MRI, and CT examination had radiation, which was not as safe as MRI [10]. Gerdes et al. [21] recommended endorectal ultrasound (EUS) as the method of choice for predicting the location of APR. But TRUS was a practitioner-dependent subjective procedure and patients would have discomfort during the examination, which was not as simple as MRI. So, we believe that the location of a rectal tumor with regard to the APR as determined by MRI is more objective and applicable.

There are some limitations in the present study that need to be pointed out. First, due to the strict inclusion and exclusion criteria, the sample size was relatively small, so some other factors (e.g., bladder filling degree, sex and uterine position) confirmed by the previous literature were not clearly related to the visualization of the APR in this study. In the future, a large-sample study should be conducted to determine whether the above factors are correlated with the visualization of the APR. Second, MRI measurement methods currently lack standardization; thus, the generalizability of findings is uncertain. Therefore, standardized measurement and external verification are required before broadening its application. Third, we did not consider whether intra-individual differences affect the visualization of APR on MRI. This aspect, partially such as patient position and intestinal peristalsis, should be deeply discussed and considered for further research.

## Conclusions

Most of the APRs are visible on MRI. Pelvic effusion, BMI, and the distance from seminal vesicle/uterus to the rectum may influence the visualization of the APR on the MRI, which is useful for evaluating the distance of AV-APR, as well as the relationship between rectal cancers and the APR. This could help clinicians in choosing the appropriate clinical decisions.

## Supplementary Information

**Additional file 1: Supplemental Table 1**. Two observers' agreement for objective parameters details.

## Data Availability

The datasets used and/or analyzed during the current study are available from the corresponding author on reasonable request.

## Abbreviations

APR: Anterior peritoneal reflection
AV: Anal verge
BMI: Body mass index
CI: Confidence interval
CT: Computed tomography
FOV: Field of view
ICC: Intraclass correlation coefficients
MRI: Magnetic resonance imaging
OR: Odd ratio
PACS: Picture Archiving and Communication Systems
SD: Standard deviation
T stage: Tumor stage
T2WI: T2-weighted image
TE: Echo time
TR: Repetition time
TRUS: Transrectal ultrasonography
