# Supplementary material for: MRI-based nomogram analysis: recognition of anterior peritoneal reflection and its relationship to rectal cancers
Source: BMC Med Imaging. 2021 Mar 17;21:50. doi: 10.1186/s12880-021-00583-7 (PMC7967971; doi:10.1186/s12880-021-00583-7)
Supplement: Supplementary file 1 — Additional file 1: Supplemental Table 1. Two observers' agreement for objective parameters details. [file 12880_2021_583_MOESM1_ESM.docx]

**Supplemental Table 1. Two observers' agreement for objective parameters details**

| Variables | | Observer 1 | Observer 2 | *Kappa* Value |
| --- | --- | --- | --- | --- |
| APR | definitely visible | 75 | 76 | 0.894 |
|  | probably visible | 35 | 34 |  |
| Degree of bladder filling | Filling | 44 | 48 | 0.889 |
|  | Not-filling | 66 | 62 |  |
| Pelvic effusion | Yes | 19 | 19 | 1.000 |
|  | No | 91 | 91 |  |
| Orientation of uterus | Anteversion | 33 | 31 | 0.892 |
|  | Retroverted | 12 | 14 |  |
| Tumor location with regard to APR (intraoperative findings) | above the APR | 16 | 17 | 0.924 |
|  | straddle the APR | 36 | 37 |  |
|  | below the APR | 58 | 56 |  |
| Tumor location with regard to APR (by MRI) | above the APR | 22 | 20 | 0.928 |
|  | straddle the APR | 40 | 41 |  |
|  | below the APR | 48 | 49 |  |

APR: anterior peritoneal reflection
